# A greater number of somatic pain sites is associated with poor mental health in adolescents: a cross-sectional study

**DOI:** 10.1186/1471-244X-13-30

**Published:** 2013-01-17

**Authors:** Shuntaro Ando, Syudo Yamasaki, Shinji Shimodera, Tsukasa Sasaki, Norihito Oshima, Toshi A Furukawa, Nozomu Asukai, Kiyoto Kasai, Yoshio Mino, Shimpei Inoue, Yuji Okazaki, Atsushi Nishida

**Affiliations:** 1 Department of Neuropsychiatry, Graduate School of Medicine, The University of Tokyo, 7-3-1 Hongo, Bunkyo-ku, Tokyo 113-8655, Japan; 2 Department of Psychiatry and Behavioral Sciences, Tokyo Metropolitan Institute of Medical Science, Kamikitazawa 2-1-6, Setagaya-ku, Tokyo 156-8506, Japan; 3 Department of Neuropsychiatry, Kochi Medical School, Kohasu Oko-cho, Nankoku, Kochi 783-8505, Japan; 4 Department of Health Education, Graduate School of Education and Office for Mental Health Support, The University of Tokyo, Tokyo, Japan; 5 Department of Cognitive-Behavioral Medicine, Kyoto University School of Public Health, Yoshida-Konoe-cho, Sakyo-ku, Kyoto 606-8501, Japan; 6 Mino Clinic, 8F Urban Office Building, Shimo-ishii 1-1-1, Kita-ku, Okayama-shi, Okayama, 700-0907, Japan; 7 Matsuzawa Hospital, Kamikitazawa 2-1-1, Setagaya-ku, Tokyo 156-0057, Japan

**Keywords:** Number, Somatic pain site, Poor mental health, Adolescents, Males and females, Academic impairment

## Abstract

**Background:**

Identifying indicators of poor mental health during adolescence is a significant public health issue. Previous studies which suggested an association between the number of somatic pains and depression have mainly focused on adults or have employed samples with a narrow age range. To date, results from previous studies have been inconsistent regarding the association between somatic pain and academic impairment. Therefore, the main aims of the present study were to 1) investigate the association between the number of somatic pain sites and poor mental health using a community sample of adolescents aged 12 to 18 years and employing a simple method of assessment, and 2) examine the association between the number of somatic pain sites and perceived academic impairment.

**Methods:**

Data analysis was conducted using a large cross-sectional survey of adolescents in grades 7 to 12. The one-month prevalence rates for three sites of somatic pain including head, neck and shoulders, and abdomen were examined. Poor mental health was evaluated using the General Health Questionnaire, and perceived academic impairment was measured using a self-report questionnaire.

**Results:**

A total of 18,104 adolescents participated in the survey. A greater number of pain sites was associated with poor mental health, and this association was consistent across age and gender. There was no difference in effect on mental health between any of the pain sites. Although there was an association between the number of somatic pain sites and perceived academic impairment, the results suggested that the association was mediated by poor mental health.

**Conclusions:**

Simple reporting methods for assessing the number of pain sites may be a feasible indicator of poor mental health in adolescents. Professionals working with adolescents should consider the possibility of poor mental health, especially when students report multiple somatic pains.

## Background

Mental health problems are a serious public health concern, especially among adolescents. Moreover, depression is the leading cause of disease burden in young people aged 10–24 years [1], with suicide the second leading cause of death within this age group, accounting for approximately 6% of total deaths [2]. However, a survey conducted in Europe demonstrated that use of psychiatric services among young people was lower than among adults [3]. This may be due to the fact that young people with depression are reluctant to seek help from professionals [4]. Thus, it is important for professionals working with adolescents to detect signs of poor mental health in youth. For the purpose of early detection, sensitive and feasible indicators of poor mental health in adolescents are required.

One indicator of poor mental health is somatic pain. Previous studies have found that such pain is a common complaint among adolescents [5,6]. A survey employing a large sample from the Netherlands showed that approximately 54% of children and adolescents had experienced somatic pain within the previous 3 months [7]. This study also demonstrated an association between somatic pain and poor mental health, as well as physical discomfort caused by somatic pain. A recent study reported that abdominal pain was associated with depressive symptoms in schoolchildren [8], while an additional study demonstrated a correlation between low back pain and depression in adolescents [9]. In addition to studies investigating single sites of pain, several previous studies have simultaneously examined multiple sites of pain. Although a linear relationship between number of pain sites and level of depression has been suggested in a few previous studies using adolescent samples [10,11], those studies used samples with relatively narrow age ranges and took into account only frequently reported pains. Because complex questions and long-term recall are required to characterize pain in more detail, including its severity, frequency, and nature, we speculated that a simpler reporting of pain might be feasible as an indicator. Therefore, the present study employed a broad age range during adolescence and a simple report of somatic pain.

A previous study demonstrated that children with somatic pain had substantial impairment in their daily lives [12]. However, the association between somatic pain and academic impairment has not been thoroughly examined. Given that school is equivalent to “work” for adolescents, that association should be a focus of investigation. Additionally, results among previous studies regarding the association between academic impairment and somatic pain have been inconsistent [13,14]. Although a previous study found that students with chronic pain experienced a decline in academic grades [13], another study reported that the level of academic competence in adolescents with chronic pain was consistent with their intellectual ability [14]. Therefore, further study examining the link between academic impairment and somatic pain is warranted.

Thus, the objectives of the present study were to 1) investigate the association between the number of somatic pain sites and poor mental health in a large sample of adolescents with a broad age range using a simple method of assessment, and 2) examine the association between the number of pain sites and perceived academic impairment.

## Methods

### Study design, sample, and survey procedures

The present study employed a cross-sectional design and used a sample of adolescent students in public junior high schools (7th–9th grades) and public high schools (10th–12th grades). The survey was conducted between 2008 and 2009. Under Japanese law, junior high school education is compulsory, but high school education is not. The present study was approved by the ethics committees of the Tokyo Institute of Psychiatry, Mie University School of Medicine, and Kochi Medical School. This research was conducted in accordance with the Helsinki Declaration as revised in 1989.

The principal investigators of the study asked all the heads and administrators of public junior highs in the city of Tsu (population 280,000) and public junior high/high schools in Kochi prefecture (population 790,000) to participate in the survey. Subsequently, the administrators and heads of schools consulted with teachers and parents to obtain their consent to participate. Instructions and guidelines for the distribution and collection of the questionnaire packets were provided to the teachers in the participating schools. For each school, teachers distributed the questionnaires to students, along with envelopes to place their surveys in when completed. All student responses remained confidential and were handled anonymously. Furthermore, teachers explained that participation in the study was strictly voluntary and assured confidentiality. Teachers reported on the total number of students who participated on the day of the survey. The research team collected the sealed envelopes from each school.

### Measures

The distributed questionnaire packets included the following items: 1) the Japanese version of the 12-item General Health Questionnaire (GHQ-12) [15]; 2) a list of three sites of somatic pain (head, neck and shoulder, and abdomen); 3) perceived academic competence; and 4) additional variables including demographic characteristics, sleeping time, history of substance use, and the experience of being bullied or being subjected to violence.

### GHQ-12

The GHQ-12 is one of the most widely used self-report measures for assessing anxiety and depression [16]. It has been used and validated in younger samples as well as in adults [17]. Additionally, previous studies have established the validity and reliability of the Japanese version of the instrument [15]. A 4-point scale using binary scoring (0011) was used for the 12 GHQ items. Responses for each question were added together to form a total score, with a range between 0 (best possible) and 12 (worst possible). We defined individuals with a total GHQ-12 score ≥4 as having poor mental health, based on findings from previous studies [17,18].

### Three sites of somatic pain

The one-month prevalence of somatic pain was assessed using a list of three main sites for pain experienced including head, neck and shoulders, and abdomen. Participants were asked to mark all the sites where they had experienced pain in the previous month. The three sites for somatic pain were chosen based on previous reports of pain with the highest prevalence among adolescents. According to these studies, headache and abdominal pain are the two most prevalent somatic pains experienced during adolescence [6,10]. Based on prevalences reported in previous studies [6,10,19], neck and shoulder pain is the third most prevalent. Participants who marked a particular site for pain were considered to have pain at that site.

### Perceived academic impairment

Perceived academic impairment was assessed using the following two questions: “Have you had difficulty concentrating on your studies recently?” “Do you feel frustrated with a recent decline in your academic performance?” The participants were asked to choose one response from the following four: “yes”, “somewhat”, “not really”, and “no”. The participants who selected “yes” were regarded as having a perceived academic impairment.

### Additional variables

One-month prevalence of alcohol use and smoking were assessed using a yes/no response format. Lifetime prevalence of any drug use was assessed using the following question: “Have you ever used any drugs?” The participants selected one of the following four responses: “no”, “only once”, “twice”, “more than three times”. Those who indicated using at least once were identified as drug users. The experience of being bullied within the past year and violence from adult cohabitants within the previous month were assessed dichotomously. Sleeping time was assessed using the following question: “Approximately how many hours do you sleep every day?” Sleeping 7 hours or less was regarded as a short sleeping time. Previous reports have shown an association between somatic pain and sleep problems such as reduced sleep [20,21]. Demographic characteristics including age, gender, and school grade were also assessed.

### Statistical analysis

One-month prevalence of somatic pains was calculated. Chi-square tests were performed to compare the prevalence of somatic pains between genders or school grades. The prevalence of each somatic pain was compared using the McNemar test. The parametric t-test was used to compare means of GHQ-12 total scores between genders or school grades.

Multivariable analysis using logistic regression modeling was performed in which the outcome of interest was poor mental health, and the exposure of interest was the number of pain sites. Variables found to have an association with both outcome and exposure were selected as possible confounders. The likelihood ratio test was performed to examine interaction between age, gender, and the number of pain sites. A similar analysis was conducted treating each pain site as the exposure of interest.

In addition, multivariable logistic regression analysis was conducted in which the outcome of interest was perceived academic impairment, and the exposure of interest was the number of pain sites. The first model included only age and gender as confounding variables. The second model included additional confounders based on findings from previous studies [20,22]. The third model included the total score for GHQ-12 to examine the effect of poor mental health on the association between somatic pains and perceived academic impairment.

## Results

The schools that agreed to participate in this study were from the following areas in Japan: 13 out of 20 public junior high schools in the city of Tsu, and 32 out of 118 public junior high schools and 28 out of 36 public high schools in Kochi prefecture. Among the 19,436 students from the participating schools, 18,638 were approached at school (798 were absent), of whom 18,250 agreed to participate in the survey. From these 18,250 participants, 18,104 responses were analyzable (a total of 93.1% of students from all the schools). Of these 18,104 students, 49.7% were male, and 50.3% were female. Their age ranged from 12 to 18 years, with a mean age of 15.2 years (SD = 1.7 years).

The one-month prevalences of each type of somatic pain for each gender and school grade are presented in Table 1. For all three somatic pains, the prevalence was higher in females than in males (p < 0.01). One-month prevalences of headache and abdominal pain were higher than that of neck and shoulder pain (p < 0.01). Regarding differences among school grades, the prevalence of neck and shoulder pain was higher in high school students than in junior high school students (p < 0.01). The prevalence of headache and abdominal pain was comparable between junior high and high school students.

**Table 1:** **One**-**month prevalence of somatic pains and the 12**-**item General Health Questionnaire** (**GHQ**-**12**) **scores by school grade**

		**Headaches**		**Neck and shoulder pain**		**Abdominal pain**		**GHQ**-**12 total score**		**Difficulty in concentrating on study**		**Frustration with poor academic performance**	
	**N**	**N**	**(%)**	**N**	**(%)**	**N**	**(%)**	**Mean**	**SD**	**N**	**(%)**	**N**	**(%)**
Demographic characteristics													
Junior high school (7^th^-9^th^)													
Males	4446	1273	(28.6)	509	(11.4)	1197	(26.9)	2.45	2.77	748	(17.0)	993	(22.6)
Females	4174	1807	(43.3)	951	(22.8)	1958	(46.9)	3.82	3.18	875	(21.1)	1185	(28.7)
Total	8620	3080	(35.7)	1460	(16.9)^a^	3155	(36.6)	3.11^b^	3.05	1623	(19.0)	2178	(25.5)
High school (10^th^ -12^th^)													
Males	4546	1215	(26.7)	630	(13.9)	1128	(24.8)	3.15	3.00	1004	(22.3)	1202	(26.6)
Females	4938	2123	(43.0)	1483	(30.0)	2066	(41.8)	4.62	3.19	1204	(24.5)	1433	(29.2)
Total	9484	3338	(35.2)	2113	(22.3)^a^	3194	(33.7)	3.92^b^	3.18	2208	(23.4)	2635	(28.0)
Total													
Males	8992	2488	(27.7)^c^	1139	(12.7)^c^	2325	(25.9)^c^	2.81^d^	2.91	1752	(19.6)	2195	(24.6)
Females	9112	3930	(43.1)^c^	2434	(26.7)^c^	4024	(44.2)^c^	4.25^d^	3.21	2079	(22.9)	2618	(29.0)
Total	18104	6418	(35.5)^e^	3573	(19.7)^e^	6349	(35.1)^e^	3.53	3.15	3831	(21.3)	4813	(26.8)

a: the prevalence of neck and shoulder pain was higher in high school students than in junior high school students (p < 0.01).

b: mean total score for the GHQ-12 was higher in high school students than in junior high school students (p < 0.01).

c: prevalence was higher in females than in males (p < 0.01).

d: mean total score for the GHQ-12 was higher in females than in males (p < 0.01).

e: the prevalence of headache and abdominal pain were higher than that of neck and shoulder pain (both p < 0.01).

Mean total score for the GHQ-12 was significantly higher in females than in males (p < 0.01). Moreover, the mean total score for the GHQ-12 was higher in high school students than in junior high students (p < 0.01).

Stratified odds ratios for each age and gender group are presented in Table 2. According to the test for interaction, there was no interaction between age and number of pain sites or gender and number of pain sites (p = 0.84 and p = 0.31, respectively).

**Table 2:** Effect of the number of pain sites on mental health by age group and gender

	**Total**	**1 pain site**		**2 pain sites**		**3 pain sites**	
	**N**	**OR**	**(95% CI)**	**OR**	**(95% CI)**	**OR**	**(95% CI)**
Age (years)^a)^							
12	720	1.88	(1.20–2.95)	3.20	(1.88–5.46)	3.97	(1.82–8.67)
13	2937	1.92	(1.55–2.38)	3.71	(2.91–4.74)	5.88	(3.94–8.77)
14	3011	1.75	(1.42–2.14)	2.57	(2.03–3.25)	4.96	(3.40–7.24)
15	3060	1.60	(1.32–1.95)	3.00	(2.39–3.76)	5.45	(3.79–7.83)
16	3438	1.76	(1.47–2.10)	3.29	(2.64–4.10)	6.46	(4.43–9.41)
17	3096	2.07	(1.72–2.50)	2.87	(2.27–3.63)	5.04	(3.44–7.39)
18	1842	2.14	(1.67–2.76)	3.28	(2.43–4.42)	5.38	(3.29–8.79)
Gender^b)^							
Males	8992	1.84	(1.64–2.06)	3.02	(2.61–3.50)	4.23	(3.28–5.47)
Females	9112	1.87	(1.67–2.10)	3.16	(2.78–3.60)	6.28	(5.13–7.69)

a) odds ratio in comparison with those with no pain sites, adjusted for alcohol use, smoking, drug use, sleeping time, experience of being bullied and violence from parents, and gender.

b) odds ratio in comparison with those with no pain sites, adjusted for alcohol use, smoking, drug use, sleeping time, experience of being bullied and violence from parents, and age.

**Figure 1:**
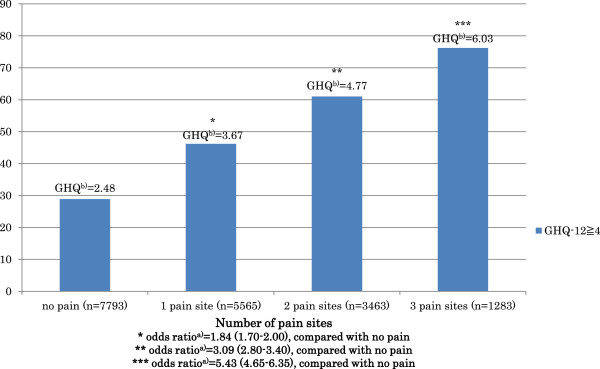
Association between number of pain sites and poor mental health.

The odds of poor mental health increased as the number of pain sites increased (Figure 1). A total of 76.2% of students who reported three pain sites had poor mental health, while less than one third of students without pain had poor mental health.

Each site of somatic pain alone increased the odds of having poor mental health (Table 3). Although the OR for neck and shoulder pain was slightly higher than for the other two pain sites, there was no significant difference among the ORs for the three pain sites. With respect to a combination of two different sites of pain, there was no significant difference in the ORs among the three different combinations.

**Table 3:** Association between site of pain and risk for poor mental health

		**GHQ-12≧4**		**Odds ratio**^**a)**^
	**N**	**N**	**%**	**(95% Confidence Interval)**
no pain	7793	2252	28.9	(reference)
headache only	2272	1015	44.7	1.80 (1.62-2.01)
neck and shoulder pain only	1000	505	50.5	1.97 (1.69-2.29)
abdominal pain only	2293	1049	45.7	1.84 (1.65-2.05)
headache and neck and shoulder pain	690	435	63.0	3.31 (2.75-3.97)
headache and abdominal pain	2173	1308	60.2	3.09 (2.76-3.47)
abdominal pain and neck and shoulder pain	600	369	61.5	2.93 (2.41-3.55)

a) odds ratio adjusted for age, gender, alcohol use, smoking, drug use, sleeping time, experience of being bullied and violence from parents.

Results showed that difficulty in concentrating on studies increased as the number of somatic pain sites increased (Table 4). However, after adjusting for GHQ-12 total score, there was no evidence of an association between the number of pain sites and difficulty in concentrating. This suggested that the effect of somatic pains on concentration when studying may have been mediated by poor mental health.

**Table 4:** Multivariable logistic regression of the effect of the number of pain sites on perceived academic impairment

	**Difficulty in concentrating**			**Frustration with poor academic performance**		
	**Adjusted OR**^**d)**^	**95% CI**	**p**-**value**	**Adjusted OR**^**d)**^	**95% CI**	**p**-**value**
Model 1^a)^						
1 pain site	1.36	(1.24–1.48)	<0.01	1.29	(1.19–1.40)	<0.01
2 pain sites	1.79	(1.62–1.98)	<0.01	1.76	(1.60–1.92)	<0.01
3 pain sites	2.66	(2.33–3.04)	<0.01	2.58	(2.27–2.92)	<0.01
Model 2^b)^						
1 pain site	1.31	(1.19–1.44)	<0.01	1.26	(1.16–1.38)	<0.01
2 pain sites	1.66	(1.49–1.85)	<0.01	1.65	(1.49–1.82)	<0.01
3 pain sites	2.23	(1.92–2.59)	<0.01	2.26	(1.96–2.60)	<0.01
Model 3^c)^						
1 pain site	1.02	(0.92–1.14)	0.65	1.04	(0.94–1.14)	0.45
2 pain sites	1.05	(0.93–1.18)	0.46	1.14	(1.03–1.27)	0.02
3 pain sites	1.14	(0.97–1.34)	0.11	1.32	(1.14–1.54)	<0.01

^a)^Adjusted for age and gender.

^b)^Adjusted for age, gender, alcohol use, drug use, smoking, sleeping time, experience of being bullied, and violence from parents.

^c)^Adjusted for age, gender, alcohol use, drug use, smoking, sleeping time, experience of being bullied, violence from parents, and General Health Questionnaire (GHQ-12) total score.

^d)^Baseline group comprised students with no reported pain.

Similarly, frustration regarding a recent decline in academic performance increased as the number of pain sites increased (Table 4). As a result of multivariate logistic regression, a substantial decrease in odds ratio was observed after adjusting for GHQ-12 total score, which also suggested a mediating effect of poor mental health.

## Discussion

The present study was one of the first using a large sample of adolescent students aged 12 to 18 years that showed a greater number of somatic pain sites associated with poor mental health. An important implication of the present study is that simple questions may be used to properly assess the three most prevalent somatic pains reported by this population. In turn, somatic pains may be feasible indicators of poor mental health in adolescents. Furthermore, the present findings suggest that the positive association between the number of pain sites and perceived academic impairment was mediated by poor mental health.

The present study did not restrict reports of pain to only those considered chronic or intense. Thus, a direct comparison with previous studies that examined chronic or frequent pain could not be made [6,7,10,19]. However, the prevalence of somatic pains in the present study was similar to that reported in previous studies. Additionally, a higher prevalence was found in females compared to males, which was in line with previous studies [7,10,19]. Moreover, a higher prevalence of somatic pains among older students was found, also consistent with previous research [7,19].

The mean total score of the GHQ-12 was similar to that found in a previous study (3.54, SD 3.04) conducted in Japan [17]. With regard to a mean difference in mental health between males and females, the present findings were consistent with past research that demonstrated a higher prevalence of emotional problems in females than in males [23]. In addition, in terms of school grade, the present study’s findings correspond with a previous study reporting that somatic symptoms increase with age [23].

The present study revealed that the association between number of pain sites and poor mental health was consistent across males and females, as well as across a broad age range during adolescence. The observed association between a greater number of pain sites and poor mental health was consistent with previous studies [10]. However, because the present study employed a cross-sectional design, a causal relationship could not be determined. To date, a bidirectional relationship between somatic pain and poor mental health has been suggested in several studies using adult samples [24,25], and there appears to be evidence supporting this relationship. In addition, social distress and physical pain reportedly have common underlying neural circuitry [26], and inflammatory mediators are potent modulators of affect [27].

Although different criteria were used to assess poor mental health, the ORs for headache and abdominal pain were similar to those found in Härmä’s study [10]. In contrast, the OR for neck and shoulder pain was higher in the present study than in that previous study. Reasons for the higher OR observed in the present study have not been identified.

The association between somatic pain and perceived academic impairment is comparable to a previous study that showed a positive association between number of pain sites and problems in daily activities [28]. Additionally, results from the present study are consistent with a study showing a correlation between a decline in academic performance scores and poor mental health [29]. Thus, the observed mediation by poor mental health suggests the following pathways: 1) somatic pain may have caused poor mental health that in turn decreased concentration on studies and induced a perceived decline in academic performance; 2) a perceived decline in academic performance caused poor mental health that induced somatic pains; 3) poor mental health induced both somatic pains and a perceived decline in academic performance; or 4) somatic pain/poor mental health induced school absence, which led to a perceived decline in academic performance. Any of the aforementioned pathways seem feasible. However, the last pathway (pathway 4) was also suggested by a previous study demonstrating that school absence was most common among children with widespread pain [30].

To the best of our knowledge, this is the first study to investigate the association between the number of pain sites and poor mental health using a large adolescent sample with a broad range of age and school grades. Furthermore, this is the first epidemiological study to investigate the association between somatic pains and perceived academic impairment using a large sample of adolescent students. Because the response rate was relatively high, the sample used is considered fairly representative of junior high and high school students within the survey area.

Results from the present study should be considered with a few limitations in mind. For example, we examined only three sites of somatic pain based on previous reports of the most prevalent types of pain and did not assess other types of somatic pain such as back or limb pain. The addition of various other somatic pains could have contributed to the comprehensiveness of the study. Additionally, because this study was based on a cross-sectional design, no causal inferences can be made regarding the association between poor mental health and the number of pain sites. Moreover, we included only students who were present at the time of the survey. If the absent students had been included and length of absence had been evaluated, the pathway between somatic pain and perceived decline in academic achievement might have been better clarified. The present study used a simple self-report measure of perceived decline in academic performance and did not examine report cards. Therefore, the present findings may not reflect actual academic performance.

The present study demonstrated that simple questions may be used to accurately identify prevalent somatic pains that are good indicators of poor mental health in adolescents. Strengths of this method are ease and speed of assessment. Because reporting on pain does not necessarily rely on the ability for long-term recall, responding is fairly easy. Furthermore, when students complain of a somatic pain, teachers and school nurses should ask about other somatic pains experienced. Irrespective of the student’s age or gender, school personnel and staff should consider depression and anxiety in students, especially when they report multiple somatic pain sites. Primary care physicians and pediatricians should respond in the same manner when encountering similar situations with adolescents. Depressive individuals with comorbid somatic pain tend to use more general medical services than mental health services compared to those without pain [31]. Inquiries about pain may also be beneficial in regard to suicide prevention because previous studies on depressive patients have demonstrated that individuals with somatic pain have a lower quality of life and higher suicidal ideation [32].

Investigations examining the specific factors that contribute to the association between the number of pain sites and poor mental health should be the focus of future scientific research. Additionally, interactions between genetic factors related to depression and anxiety as well as environmental factors should be examined. Prospective studies are recommended to investigate the causal relationship between poor mental health and the number of pain sites. In addition, questions representing a more comprehensive approach to assessing somatic pains may be beneficial.

## Conclusions

Simple reporting methods for assessing number of pain sites may be a feasible indicator of poor mental health in adolescents. Professionals working with adolescents should consider the possibility of poor mental health, especially when students report multiple somatic pains. Furthermore, the association between a greater number of pain sites and perceived academic impairment can be mediated by poor mental health.

## Competing interests

All coauthors declare that they have no competing interests.

## Authors’ contributions

SA, SY, and AN designed the study and analyzed the data. SS and YO supervised the study. SA wrote the first draft of the manuscript, and all other authors were involved in revising the draft and approving the final manuscript.

## Authors’ information

Shuntaro Ando and Syudo Yamasaki are joint first authors.

## Pre-publication history

The pre-publication history for this paper can be accessed here:

http://www.biomedcentral.com/1471-244X/13/30/prepub
